# Development of a Precision Medicine Workflow in Hematological Cancers, Aalborg University Hospital, Denmark

**DOI:** 10.3390/cancers12020312

**Published:** 2020-01-29

**Authors:** Julie S. Bødker, Mads Sønderkær, Charles Vesteghem, Alexander Schmitz, Rasmus F. Brøndum, Mia Sommer, Anne S. Rytter, Marlene M. Nielsen, Jakob Madsen, Paw Jensen, Inge S. Pedersen, Lykke Grubach, Marianne T. Severinsen, Anne S. Roug, Tarec C. El-Galaly, Karen Dybkær, Martin Bøgsted

**Affiliations:** 1 Department of Haematology, Clinical Cancer Research Centre, Aalborg University Hospital, Sdr. Skovvej 15, DK-9000 Aalborg, Denmark; j.boedker@rn.dk (J.S.B.); mads.soenderkaer@rn.dk (M.S.); martin.boegsted@rn.dk (M.B.); 2 Department of Clinical Medicine, Aalborg University, Sdr. Skovvej 15, DK-9000 Aalborg, Denmark; 3 Clinical Nursing Research Unit, Aalborg University Hospital, Sdr. Skovvej 15, DK-9000 Aalborg, Denmark; 4 Department of Molecular Diagnostics, Aalborg University Hospital, Reberbansgade 15, DK-9000 Aalborg, Denmark; 5 Department of Pathology, Aalborg University Hospital, Ladegårdsgade 3, DK-9000 Aalborg, Denmark

**Keywords:** precision medicine, hematology, next generation sequencing, somatic cancer variants, variant interpretation, bioinformatics workflow

## Abstract

Within recent years, many precision cancer medicine initiatives have been developed. Most of these have focused on solid cancers, while the potential of precision medicine for patients with hematological malignancies, especially in the relapse situation, are less elucidated. Here, we present a demographic unbiased and observational prospective study at Aalborg University Hospital Denmark, referral site for 10% of the Danish population. We developed a hematological precision medicine workflow based on sequencing analysis of whole exome tumor DNA and RNA. All steps involved are outlined in detail, illustrating how the developed workflow can provide relevant molecular information to multidisciplinary teams. A group of 174 hematological patients with progressive disease or relapse was included in a non-interventional and population-based study, of which 92 patient samples were sequenced. Based on analysis of small nucleotide variants, copy number variants, and fusion transcripts, we found variants with potential and strong clinical relevance in 62% and 9.5% of the patients, respectively. The most frequently mutated genes in individual disease entities were in concordance with previous studies. We did not find tumor mutational burden or micro satellite instability to be informative in our hematologic patient cohort.

## 1. Introduction

With the emergence of affordable next generation sequencing, an increasing understanding of oncogenic driver mechanisms, and development of drugs targeting specific molecular changes, many precision medicine initiatives worldwide have been initiated [1,2]. The overall goal in precision cancer medicine is to match patients to treatments with higher efficacy using genomic information from the patient’s tumor [3]. This requires detection of somatic alterations and linking these to dysregulation of oncogenic genes or pathways, that in turn leads to sensitivity or resistance to cancer treatment agents or provide information on the prognosis of the patient. Precision cancer medicine needs to be performed in an unbiased manner, since the genomic landscapes of different cancers, and hence the relevant genetic biomarkers differ widely [4,5,6,7,8,9]. The number of known molecular targets and mechanisms evaluated have increased quickly, as methods have moved from single gene analysis to panel sequencing, Whole Exome Sequencing (WES), and/or Whole Genome Sequencing (WGS) in combination with global RNA-sequencing [10]. The molecular targets taken into consideration up till today have focused on cancer related DNA and RNA changes such as small nucleotide variants (SNV), fusion genes, translocations, copy number alterations (CNA), mutational signature patterns, gene expression levels, and pathway analysis. The identified genomic variants can refine or guide the diagnosis and provide information on prognosis in e.g., hematological malignancies [11]. High levels of micro satellite instability (MSI) of the tumor guide treatment with immune checkpoint inhibitors in some solid tumors, e.g., in metastatic colorectal carcinoma [12]. Tumor mutational burden (TMB) status appears to be able to stratify these patients according to the likelihood of response [13]. A subset of patients across various cancer types displays a high TMB, and could potentially benefit from immunotherapy [14,15]. The mutational signature of somatic variants in a tumor reveals the underlying development of the cancer and can influence choice of therapy [14]. In a few cases, the evidence of clinical relevance of a genetic alteration is strong, e.g., by a link directly to a treatment guideline recommending a specific treatment towards the alteration or the genetic alteration is an inclusion criteria for a clinical trial [16]. However, the majority of identified tumor specific molecular targets has an unknown or only in silico predicted effect on the corresponding protein. The type and strength of evidence for the clinical relevance need to be carefully evaluated by the treating physician, when treatment choice is recommended.

Within the past five years, several interventional clinical studies based on genomic tumor profiling have been launched to assess the efficacy of precision medicine using end point parameters such as overall response rate, overall survival, and/or progression-free survival of cancer patients, as reviewed by Zimmer and colleagues [17]. Some studies have included patients with refractory solid cancers, but only a few studies have included patients with hematological malignancies [18,19,20]. Galanina and colleagues identified molecular alterations targetable with gene- or immune-targeted agents using targeted sequencing in 227 hematological cancer patients [20] and Laganà and colleagues presented a precision cancer medicine initiative of relapsed and/or refractory multiple myeloma (MM) patients based on DNA and RNA sequencing [21]. The study by Mody and colleagues is to our knowledge the only one using WES in combination with RNA-sequencing for the genomic profiling of refractory or relapsed solid and hematological cancer patients, but their study only included children and young adults [19]. Hence, little is known about the potential of DNA exome and RNA sequencing initiatives of precision medicine in adult patients with progression of or relapsed hematological malignancies. 

Here, we explore the potential of a precision medicine initiative based on WES and RNA sequencing in hematological patients with progressive disease or newly verified relapse. We present the hematological precision medicine workflow developed at Aalborg University Hospital in a prospective, consecutively, and therefore unbiased population-based non-interventional study including 174 patients enrolled between 2016 and 2019, of which 92 samples were sequenced. We elaborate on the developed logistics to enroll patients, collection of tumor/normal samples, laboratory choices, database and laboratory information management systems, the bioinformatics workflow, and the curation of identified genomic variants with a match to treatments or clinical trials. Moreover, we also investigate the clinical relevance of TMB, MSI status, and mutational signature patterns in hematological malignancies. 

## 2. Results

### 2.1. Patient Screening, Enrolment, and Sample Processing

**Figure 1:**
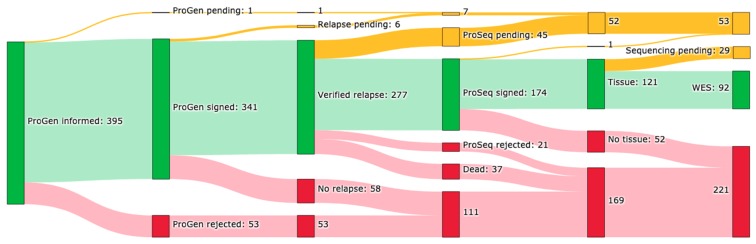
Inclusion overview. Number of patient cases included in the ProGen and ProSeq studies by 31 January 2019. Patient’s relapse status, presence of correct tumor tissue for analysis, and sample status is given. Color codes: green: yes, yellow: pending, and red: no.

In total, 588 patient visits have been screened for potential participants to the ProGen protocol, Figure 1. In 395 (67%) of these visits, patients were offered enrollment, which was accepted in 341 (86%) cases. In 277 (81%) of these cases, the patient had a verified clinical relapse or progressive disease. Since 16 February 2017, enrolment in the ProSeq protocol was offered in 271 cases to patients with verified relapse. Of these, 174 (64%) were enrolled, 45 (17%) are pending, 21 (8%) rejected enrollment, and 37 (14%) received study information, but died before enrollment. In 52 cases (30%), biological material suitable for sequencing analysis was missing. By 31 January 2019, 92 tumor samples (76%), from 85 patients were sequenced, analyzed, and evaluated, Figure 1.

### 2.2. Patient Characteristics 

In total, 92 genomic profiles from 85 different patients with 18 different hematological diagnoses were stratified into six different hematological diagnosis groups, Table 1. Five patients had two and a single patient had three genomic profiles from different relapses. The median age was 70 years old (range 27–92), and the patient cohort had a slight majority of male patients (60%).

### 2.3. DNA and RNA Purification from Different Flow Sorted Cell Types

Differences in DNA yield were small between multi parameter flow cytometry (MFC) sorted cells from different disease entities (max = 1.6 fold) or sample types (max = 1.1 fold). As expected, for RNA yield, samples originating from patients with plasma cell disease had a median yield of up to 5.4 fold larger than any other disease group, while an insignificant difference in yield was observed when comparing sample types, Figure S1. 

### 2.4. Sequencing Quality Control

**Figure 2:**
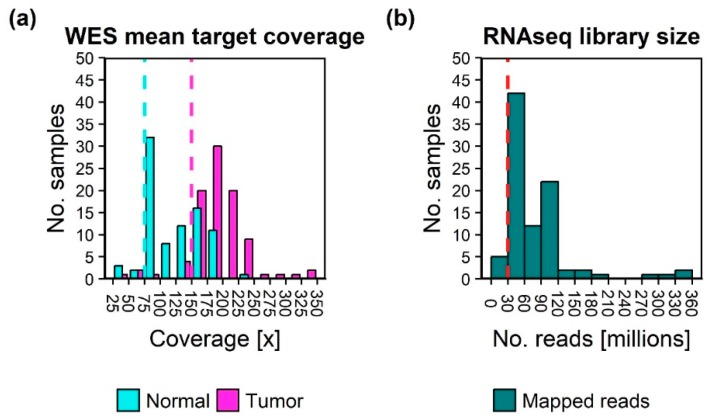
Histograms of sample sequencing depth. (**a**) Average target coverage of normal and tumor Whole Exome Sequencing (WES) samples. (**b**) Total mapped paired end RNAseq reads. Dashed lines indicate the minimum sample depth requirements.

In total, 92 tumors with corresponding 85 normal samples were whole exome sequenced. All but one had a corresponding tumor RNAseq sample. The desired minimum average coverage of 75× and 150× for normal and tumor WES samples, respectively, and 30M sequenced reads for RNAseq libraries enabled processing of two patient sample sets pr. sequencing run on an Illumina NextSeq500 sequencer, which was available in-house. With a mean sequence coverage of 150×, the false negative rate of an alteration with an allele frequency of 10% would be less than 0.055% (the probability based on the binomial distribution that the alteration would be observed with less than 5 reads). Normal and tumor WES samples were sequenced to a median of sample mean coverages of 117× (range 31–234) and 185× (range 47–331), respectively, Figure 2. Most samples were well above the minimal requirement. RNAseq samples contained between 26.7 and 354 million mapped reads (median 52.7 million). The average target coverage was below the minimum sample depth for five normal samples (5.8%), eight tumor samples (8.7%), and five RNAseq samples (5.3%). To allow evaluation on a true prospective collected population, samples were still included in downstream analysis, keeping the lower coverage in mind during variant analysis.

### 2.5. Variant Classification

**Figure 3:**
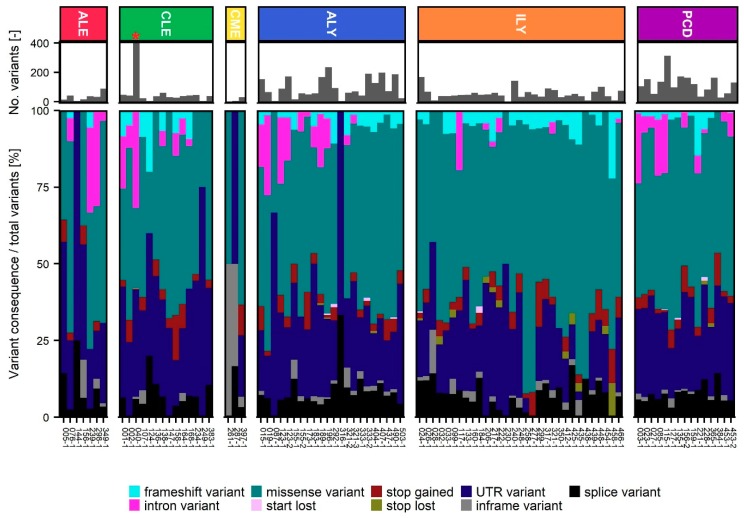
Small nucleotide variant (SNV) detection and filtering. Retained variants per patient grouped by type and divided into the six disease groups; ALE: Acute leukemia, CLE: Chronic leukemia, CME: Chronic Myeloid Neoplasms, ALY: Aggressive Lymphomas, ILY: Indolent Lymphomas, and PCD: Plasma Cell Diseases. * Sample contained 5061 variants and was not subjected to variant interpretation in Qiagen Clinical Insight Interpret.

The median number of SNV was 572 per WES tumor sample (range 78–17,256). Following additional filtering based primarily on variant consequence and population frequencies, 14% of detected variants were on average retained for variant evaluation (range 0.2–66%). Samples from aggressive lymphomas and plasma cell disease had a significantly higher amount of retained variants compared to each of the four other diagnosis groups (*p*-value < 0.05). The number of variants and the relative number of retained variant types in each sequenced tumor sample, grouped by disease entities, can be seen in Figure 3.

**Figure 4:**
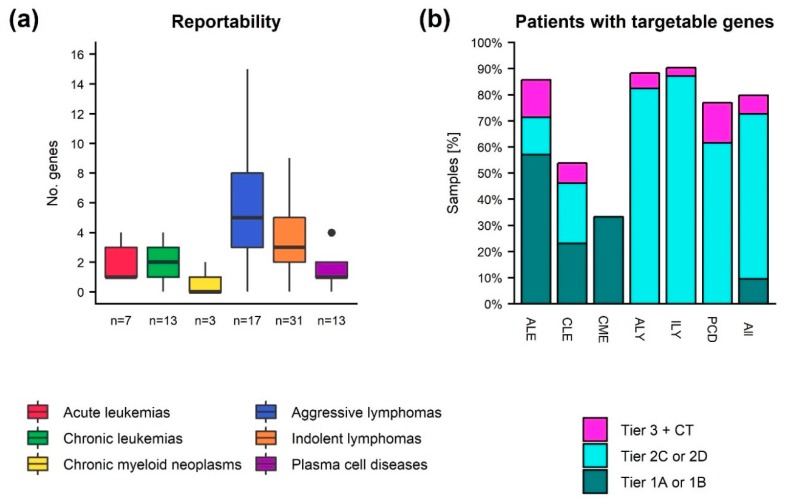
Reportable somatic variants in hematological malignancies. (**a**) Number of genes with detected somatic variants reported per sample after manual verification. (**b**) Percentage of patients (grouped by diagnosis or overall) with at least one variant having strong clinical relevance (tier 1A and 1B), potential clinical relevance (tier 2C and 2D), or at most having unknown clinical significance, but with an associated clinical trial (tier 3 + CT). If multiple samples were sequenced for a patient, only the latest is represented in the figure. The single sample from a patient with CLL with more than 5000 retained variants was not subjected to variant interpretation. ALE: Acute leukemia, CLE: Chronic leukemia, CME: Chronic Myeloid Neoplasms, ALY: Aggressive Lymphomas, ILY: Indolent Lymphomas, PCD: Plasma Cell Diseases.

Variant interpretation was conducted in 91 cases from 84 different patients, Table S1. On average, somatic variants were reported in 3.3 genes (range 0–15) due to either their therapeutic, pathogenic, or prognostic clinical relevance, Figure 4. At least one somatic variant was reported in 81 cases (89%). Variants with strong clinical significance (tier 1) were reported in eight patients (9.5%), Figure 4. Of these, six patients had variants that were targets for FDA- or EMA-approved clinical therapies and two had variants with prognostic value included in professional guidelines. Variants with strong therapeutic significance were found in either *IDH1*, *IDH2*, or *TP53* in three different patients diagnosed with acute myeloid leukemia (AML) or in *TP53* in three patients diagnosed with chronic lymphocytic leukemia (CLL). In two cases, loss of function variants in *RUNX1* with strong prognostic significance were detected. All eight patients with tier 1 variants also had tier 2 variants that served as an inclusion criterion for one or more clinical trials.

**Figure 5:**
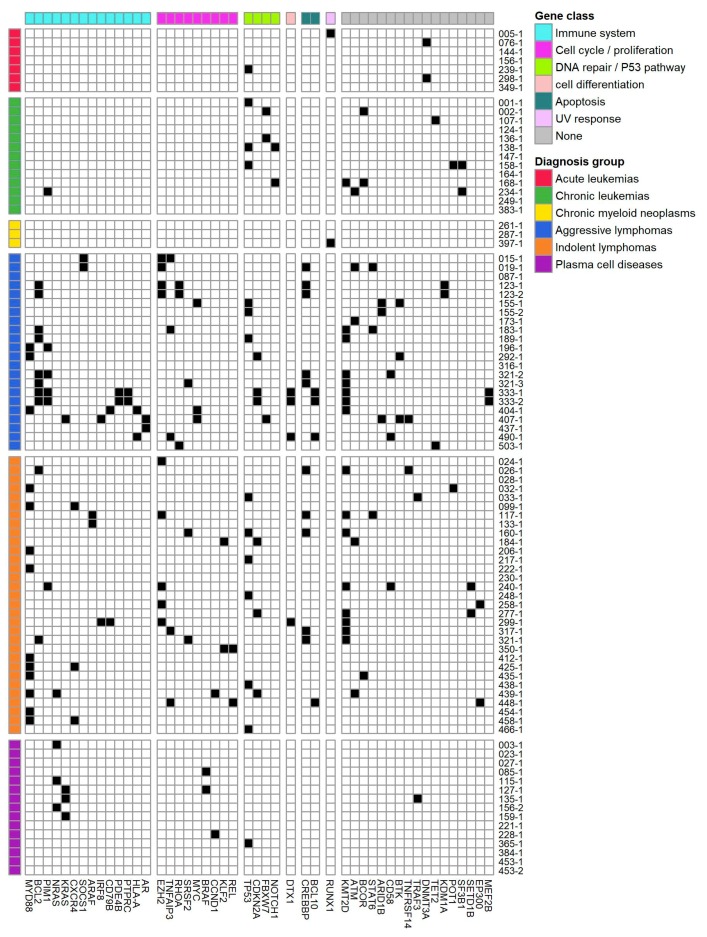
Occurrence of genes with clinically relevant alterations. The genomic landscape of distinct, clinically relevant gene alterations across various hematologic cancers if observed in more than one patient. Each row represents a patient sample. These are grouped by diagnosis group. Each column represents gene with clinical relevant alterations. Genes are organized by gene sets derived from MSigDB Collection2 (Version 6.2) [23,24,25].

Additionally, 53 patients (62%) lacking tier 1 variants had at least one variant with potential clinical relevance (tier 2), Figure 4. Of these, 29 had potential relevant therapeutic variants, which were associated with resistance or sensitivity to FDA or EMA approved clinical therapies for a different diagnosis, and 48 patients had variants that served as an inclusion criterion for one or more clinical trials. Tier 2 therapeutic relevant variants where found in seven different well-known cancer genes [22] (no. patients given in parenthesis): *TP53* (8), *EZH2* (8) *KRAS* (4), *NRAS* (3), *TET2* (2), *SRSF2* (1), *NOTCH1* (1), and *BRAF* (1). Overall, 11,857 different SNVs from 7250 different genes were subjected to variant interpretation. Only 47 variants (0.3%) were detected in multiple patients, while 34% of genes with reported variants were observed in > 1 patient. Variants interpreted to be clinical relevant were found in 136 different genes. Of these genes, 44 appeared in > 1 patient, Figure 5.

Fusion genes were reported in 14 cases (17%). In most cases, the fusion genes were classified as unknown clinical relevance (tier 3), but were reported due to a potential pathogenic effect, caused by one of the fusion gene partners being a known oncogene. However, a few had potential clinical relevance. Three fusion genes, *CIITA*-*BCL6*, *MBNL1*-*BCL6*, and *CUX1*-*BRAF*, served as inclusion criteria for one or more clinical trials, and an AML patient had a *NUP98-NSD1* gene fusion indicative of poor prognosis [26].

In ten different patients (12%), 14 gene losses or amplifications were reported. Most CNA had uncertain clinical relevance (tier 3). Only two CNA had potential clinical relevance (tier 2), namely *MTAP* or *CDKN2A* loss. Both of these served as inclusion criteria for one or more clinical trials, and the latter also showed plausible resistance in a case study [27]. 

**Figure 6:**
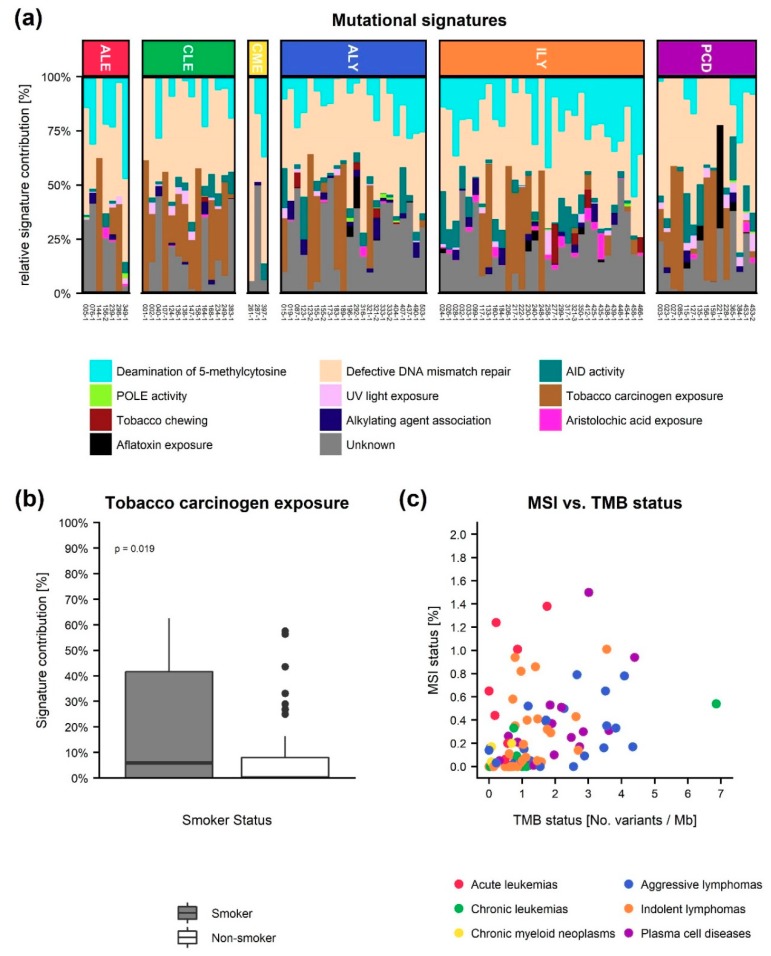
Other potential clinically relevant genomic measurements. (**a**) The relative contribution of the COMSIC mutational signatures to each tumor sample grouped as either Deamination of 5−methylcytosine, defective DNA mismatch repair, activation-induced cytidine deaminase (AID) activity, DNA polymerase epsilon (POLE) activity, UV light exposure, tobacco carcinogen exposure, tobacco chewing, alkylating agent association, aristolochic acid exposure, or aflatoxin exposure according to their proposed aetiology. ALE: Acute leukemia, CLE: Chronic leukemia, CMNE: Chronic Myeloid Neoplasms, ALY: Aggressive Lymphomas, ILY: Indolent Lymphomas and PCD: Plasma Cell Diseases. (**b**) Comparison of the relative contribution of the tobacco carcinogen exposure signature in the smoker vs. non-smoker groups. (**c**) Micro satellite instability (MSI) status vs. tumor mutational burden (TMB) status.

In addition to detection of clinically relevant somatic mutations, other genomic measures with potential clinical relevance were measured, Figure 6. None of our cases had more than 2% instable microsatellite sites. In line with this, the TMB score was also low in all cases, and the MSI/TMB ratio was not found to be significantly different between diagnosis groups. Only in a single case a TMB score > 5 variants/Mb could be detected.

The contribution of the COSMIC mutational signatures was investigated in order to detect potential signals in regards to, e.g., defective DNA mismatch repair, which is highly relevant in the light of immunotherapy treatments, Figure 6. In two patients, we identified a high amount of mutations associated to defective DNA mismatch repair, and surprisingly one of these patients did not have reported SNV. However, no significant correlation between the contribution of defective DNA mismatch repair signatures and MSI or diagnosis group could be found. When possible, we collected patient data on smoking habits (*n* = 82), Figure 6. Here, a comparison between non-smokers and current or former smokers revealed an increased relative contribution of the tobacco carcinogen exposure signature (*p*-value = 0.039, Student’s *t*-test) in the current/former smokers group.

## 3. Discussion

This study outlines in detail all steps involved in setting up the logistic, laboratory, and bioinformatics workflow of a hematological precision medicine initiative developed at Aalborg University Hospital. We chose to utilize WES and RNAseq as the basis for our genomic analyses, facilitating an unbiased and exploratory survey of the entire exome. This enabled us to discover cancer specific alterations in all regions of the exome, which can provide relevant molecular information to a multidisciplinary team. 

We reported somatic variants in 136 different genes, of which 44 genes were altered in more than one patient. Overall our findings are in line with the genomic landscape of somatic alterations found in larger hematological genomic studies [28,29,30,31], even though some hematological subtypes are only represented by a few patients in our cohort. In detail, the six most frequently altered genes in the entire patient cohort were (in descending order) *KMT2D*, *TP53*, *MYD88*, *BCL2*, *CREBBP*, and *EZH2*, which were altered in between 19 and 11% of all cases. All these are well-known cancer driver genes often mutated in lymphomas, which represented the largest proportion of patients in our cohort. Of the recurrently altered genes in our cohort, we identified variants in *TP53*, *RUNX1*, and *DNMT3A* in our small cohort of acute leukemias, Figure 5, which are known to be frequently mutated in AML [28]. For the aggressive lymphomas, the majority of the nine genes mutated in three or more patients, were also identified as frequently mutated in diffuse large B-cell lymphoma (DLBCL) by Reddy and colleagues [32] and by Karube and colleagues [6]. Four of the ten genes observed in the chronic leukemias group were also found by Burns and colleagues to be frequently mutated in CLL [30]. For the indolent lymphomas, the patients with Waldenström’s macroglobulinemia all had the hallmark *MYD88* mutation, and three of those also had a *CXCR4* mutation, both very frequent mutations in this disease [29]. In patients with follicular lymphoma (FL), we, as others [33], identified frequent mutations in *KMT2D*, *CREBBP*, and *EZH2* with frequencies of 8/10, 5/10, and 4/10, respectively. In agreement with Lohr and colleagues, we observed *NRAS*, *KRAS*, and *BRAF* to be the most frequently mutated genes in plasma cell disease [7]. The group of chronic myeloid neoplasms only included three patients, and only in one patient, a recurrent clinically relevant mutation in *RUNX1* was observed. In a study by Haferlach and colleagues, the *RUNX1* gene was mutated in around 10% of patients with myelodysplastic syndrome [31]. 

In our precision medicine analysis, we found a significantly higher number of retained variants in the aggressive lymphomas and plasma cell disease groups. This could indicate that tumor cells from relapsed patients of aggressive cancer types have obtained more somatic mutations than tumors from chronic/indolent cancer types, or that malignancies, which originate in more mature B-cells have a higher load of retained variants compared to leukemias and myeloid derived neoplasms. In the majority (71%) of the 84 patients investigated, we identified clinically relevant somatic variants (tier 1 or 2). In 42% of cases, potential relevant therapeutic variants associated with resistance or sensitivity to FDA- or EMA-approved clinical therapies were detected. In six patients, variants with strong clinical therapeutic relevance were found. These were either gain of function alterations in *IDH1* or *IDH2*, which could indicate sensitivity towards IDH inhibitors, or gain of function alterations in *TP53*, which could indicate sensitivity towards the *BCL2* inhibitor Venetoclax and sensitivity towards the kinase inhibitor Idelalisib. Patients harboring variants with strong clinical therapeutic relevance were either diagnosed with AML or CLL, indicating that the treatment potential of precision medicine in hematology greatly varies between diagnose entities. Variants with potential clinical therapeutic relevance were found in seven different well-known cancer genes, potentially giving rise to treatment. For instance, point mutations leading to *EZH2* Y641 substitutions, which are gain of function alterations of *EZH2*, were found in both DLBCL and FL patients, potentially giving rise to treatment with *EZH2* inhibitors such as Tazemetostat [5,34]. A patient with AITL and a patient with CLL had loss of function *TET2* mutations, which indicates sensitivity towards DNMT inhibitors such as Decitabine or Azacitidine, which both are tested in clinical trials for treatment in different lymphoma sub types [34]. In six patients diagnosed with MM, we found gain of function mutations in *KRAS* or *NRAS*, which are inclusion criteria for a clinical study (NCT03091257) of treatment with the *BRAF* inhibitor Dabrafenib and/or the *MEK* inhibitor Trametinib in patients with relapsed and/or refractory MM. In three patients diagnosed with CLL or SLL we found loss of function mutations of the *NOTCH1* gene. These alterations are indicative of resistance to treatment with Rituximab, but serves as an inclusion criterion for a clinical study (NCT03204188) investigating the combination of Ibrutinib, Fludarabine, and Pembrolizumab for treatment of CLL. The above examples of detected genetic alterations with clinical therapeutic relevance show the diversity of treatment options and complexity of hematological malignancies. This highlights the necessity for variant interpretation support tools, which automatically links genetic alterations with the most current clinical information available.

In 64% of cases, variants that potentially served as an inclusion criterion for one or more clinical trials were detected. This is in line with previous studies of precision medicine within hematology. Bryce and colleagues e.g., found, actionable alterations in 66% of patients (both solid tumors and hematological malignancies) [18], and a recent study by Galanina and colleagues where 227 hematological patients were sequenced, a possible drug based on molecular alterations were identified in 75% of patients [20]. However, the results might not be directly comparable, since we have slight differences in the utilized methodology and hence detection limits, and different ratios of the individual hematological malignancies, where the number of somatic alterations differ greatly between patients and diagnosis groups, Figure 3. In our patient population, all have a newly verified relapse or progression of disease at time of biological sampling. These sampling details are not included in the study by Galanina and colleagues [20]. Our study was observational, and hence did not include treatment intervention. Barriers to deliver targeted treatment include drug availability, patient performance and comorbidities, and distance to sites of potential clinical trial sites, as experienced in other precision cancer medicine initiative studies [11,18,35,36]. Most patients within our cohort were at time of sampling still eligible for guideline recommended treatment, as there are multiple different treatment regimens for most hematological diagnoses. Hence, the target group for a precision medicine initiative within hematology (early diagnosed, relapsed, or patients with no other treatment offers) needs to be determined in future initiatives.

Biomarkers such as TMB, MSI, and mutational signature status have been found to be informative across cancer types, but is mostly investigated within solid tumors [13,14]. In a study with over 7000 cancer patients, Samstein and colleagues found that diagnosis entity dependent high TMB status were associated with improved overall survival [13]. In hematological cancers, TMB levels are low compared to solid tumors, with TMB level of leukemias and myeloid neoplasms being lowest, followed by MM, mantle cell, and T-cell lymphomas, and the highest TMB is found in DLBCL and FL [14,15]. Our results are in line with these studies. In general, we also found the highest TMB levels in hematological patients with MM, FL, or DLBCL. However, a single CLL patient had a 6-fold higher TMB level than others in the group. In agreement with previous publications, we did not identify high MSI status in any of our 92 hematological tumors [37,38]. The mutational signatures of a cancer sample can guide the choice of immunotherapy if the signature displays a DNA repair deficiency profile [39]. Both MSI and the abundance of defective DNA mismatch repair related signatures might be indications of cancer where immunotherapy would be usable treatment options. However, we found no correlation between the abundance of defective DNA mismatch repair related signatures and MSI. The significant difference in the abundance of the tobacco carcinogen exposure signature between smokers and non-smokers indicates that mutational signatures could have a potential as an informative measure for hematological patients. However, additional studies are needed to elucidate the potential of mutational signatures in precision medicine of hematological cancers.

Time to clinical answer is a major issue for some hematological patients. A realistic time frame in an implemented clinical setting of the presented initiative would be 3–5 weeks. However, relapsed hematological patients often cannot wait this long before treatment initiation. Stringent and correct tissue sampling, which is an obvious necessity, can be challenging in a clinical routine setting. Of the 174 patients enrolled, 54 patients did not have the correct tissue donated for sequencing analysis. Bone marrow aspirates and lymph node biopsies were only collected to the study, if the particular tissue type was needed in the clinical evaluation of the suspected disease progression. In other cases, e.g., MM patients, the donated bone marrow did not contain enough malignant cells to perform sequencing, why a re-sampling would be a necessity—costing precious time. This could be circumvented by collecting more bone marrow at the first visit. 

The choice of WES and RNAseq as methods for our genomic analyses, was based on a desire of the analysis to be as unbiased as possible, even though panel sequencing would give a higher detection limit, and the ability to detect larger structural variants such as CNA and translocations. For detection of translocations, we utilized detection of fusion transcripts by RNAseq. This is obviously a suboptimal method since only breakpoints within gene regions can be detected. Therefore, we see detection of translocations using RNAseq as a complementary method to existing cytogenetic assays such as karyotyping and fluorescence in situ hybridization. Moreover, RNAseq data can provide information on gene expression levels. Others have found this informative in genomic cancer profiling and provided treatment recommendations based on gene expression profiles [21] and predictions of patient response to treatment agents [40,41,42]. However, these methods are based on and validated in larger microarray expression datasets of diagnostic samples. Currently, there is a lack of RNAseq based gene expression datasets originating from hematological malignant samples with corresponding clinical information. These could be used to determine a reference gene expression level in tumor samples for determination of differential expression, and validation cohorts for treatment response prediction algorithms. However, we see a great potential of implementing these methods in future precision medicine initiatives. Likewise, detection of CNA by the use of WES data is uncertain. A shift to WGS of the sample would improve the detection of translocation breakpoints often spread across several hundred kilobases and improve the detection of CNAs due to the more even sequence coverage across the entire genome. Time would also be saved, as the sequence capture step in library preparation would disappear. However, besides the added cost of sequencing, this would also require access to or purchase of a more powerful high performance computer environment, adding both cost and time for bioinformatical processing. We estimate that the data pre-processing step from raw sequence data to a set of filtered variants for biological interpretation using WES and RNAseq data can be performed in less than 24 h [43]. The same requirement would apply to WGS data pre-processing, but this would require optimization of the bioinformatical workflow, e.g., in regards to input/output options and data-level parallelization. Following data pre-processing, the manual evaluation of each somatic variant according to clinical guidelines is the next major time consuming step [44,45]. Variant interpretation is a major challenge for precision medicine in somatic cancers [46]. Only a few hundred DNA variants are well annotated as cancer drivers and targetable with drugs [22,47,48]. The vast majority of somatic variants can still only be classified as “variants of unknown significance” or has conflicting literature information on cancer relevance. This call for data sharing initiatives, to improve cancer related public databases and provide information to automated classification of variants. Within the past few years, several large data sharing initiatives have emerged which have the potential to overcome this challenge [49]. The interactive knowledge system Genomic Data Commons (GDC), from The National Cancer Institute, is a good example. Here, the goal is to democratize the access to cancer genomic data and facilitate sharing of these data. The GDC contains large hematologic data sets, e.g., nearly 2000 AML cases and more than 1000 ALL cases. However, the case number for other hematologic sub types is still low. Although data sharing initiatives exists, challenges such as patient’s trust in sharing their clinical and genomic data for research [50] and regulations of data privacy such as the EU general data protection regulation [51] needs to be met, for hematologic precision medicine to become a success.

## 4. Materials and Methods 

A detailed version of the materials and methods section is available in Appendix A.

### 4.1. Study Protocols, Screening, and Inclusion

Patients from the Department of Hematology, Aalborg University Hospital, Denmark with suspected progression or relapse of a hematological malignancy were offered participation in the ProGen (N-20150042) and ProSeq (N-20160089) studies approved by the North Denmark Region Committee on Health Research Ethics. Participants gave written informed consent in accordance with the Declaration of Helsinki. ProGen included single gene analysis. Patients were enrolled in ProGen from 1 February 2016. ProSeq included WES and RNA sequencing, and patients were enrolled from 16 February 2017. All included patients in this study were enrolled before 1 February 2019. Patients were offered participation at every suspected relapse or progression of disease in the project period, and hence could be enrolled multiple times. Both studies were observational and non-interventional studies. 

### 4.2. Biobank, Cell Sorting, and Purification

Tumor tissue included lymph node biopsies, peripheral blood, and bone marrow and as germline DNA source saliva or mouth swabs were obtained. When the cancer cell frequency was below 80%, we used multi parameter flow cytometry (MFC) and sorting with selected panels from the “Euroflow antibody panel list” [52] to purify the bulk of malignant cells from peripheral blood and bone marrow samples, Table A1. DNA and RNA were purified from vital frozen mononuclear cells, flow sorted malignant cells, or homogenized biopsies using Qiagen’s All Prep DNA/RNA/miRNA kit, following the manufacturer’s guidelines. DNA from saliva and mouth swabs were purified using the kits: PrepIT•L2P (DNA Genotek, Ottawa, Canada), and DNeasy Blood & Tissue Kit (Qiagen, Germantown, MD, USA), respectively, following the manufacturer’s guidelines. Preparation of WES and RNA libraries were performed, followed by Illumina paired end sequencing producing minimum 26 Gb, 18 Gb, or 33 million reads of raw sequence data for tumor DNA, normal DNA, or tumor RNA samples, respectively.

### 4.3. Bioinformatics Workflow

**Figure 7:**
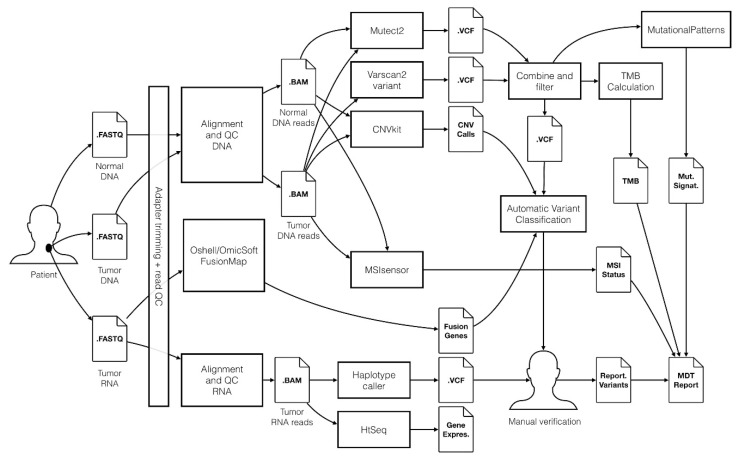
Overview of the bioinformatics workflow for processing of WES and RNA sequencing data in the ProSeq study. The workflow is based on the Genome Analysis Tool Kit (GATK) framework, and Qiagen Clinical Insight Interpret is used to support the manual variant interpretation performed by molecular biologists. Genomic data results are stored in a precision cancer medicine database, potentially enabling results to be shared with other hospital organizations and the scientific community.

We developed an in-house bioinformatics workflow for data processing and analysis, Figure 7. For a detailed description, see the online Supplementary Material. The workflow utilizes the Genome Analysis Tool Kit (GATK) v3.8 framework [53], predominantly following the GATK best practices [54,55]. Additionally, using WES data CNA, TMB, MSI status, and mutational signature analyses were performed. Fusion transcript detection was performed using RNAseq data. Finally, detected somatic variants, along with patient information (age, gender, and diagnosis) were uploaded to Qiagen Clinical Insight Interpret (www.qiagenbioinformatics.com) for automatic classification. Classification was performed in an unbiased manner, where each detected variant was classified by pathogenicity as either pathogen, likely pathogen, unknown, or benign and by clinically actionability as either having strong (tier 1), potential (tier 2), or unknown (tier 3) clinical relevance based on its therapeutic, prognostic, or diagnostic potential. The evaluation was based on, current clinical evidence from drug labels, clinical guidelines (e.g., NCCN [56], ASCO [57], and ESMO [58]), and clinical trials at clinicaltrials.gov according to the 2015 ACMG/AMP [45] and the 2017 AMP/ASCO/CAP guidelines [16]. Final manual variant curation was performed by two trained molecular biologists. To enable workflow monitoring and facilitate the FAIR data principles [59,60], a laboratory information management systems solution based on the REDCap application was developed [61,62].

## 5. Conclusions

This demographic, unbiased, observational, and prospective precision cancer medicine study included hematological patients with disease progression or relapse at Aalborg University Hospital, Denmark. The analysis workflow was based on WES and RNA sequencing and results of 92 patient samples from 85 patients are presented here. In conclusion, the most frequently mutated genes in individual disease entities were in concordance with previous studies. In our hematologic patient cohort, TMB and MSI were not found to be informative. Based on analysis of small nucleotide variants, copy number variants, and fusion transcripts, we found variants with potential and strong clinical relevance in 62% and 9.5% of the hematological patients, respectively. The developed workflow provides genomic information ready for interpretation by the treating clinician or a multidisciplinary team, who can create an overview of the clinical and genomic status of the patient and decide if, e.g., off label treatment or enrolment in a clinical trial is a possibility for the patient. The patient group in our study was hematological patients, but the workflow can easily be extended to other cancer types where tumor tissue is available and DNA/RNA can be extracted.

## Figures and Tables

**Table 1:** Patient characteristics per diagnosis group.

Diagnosis Group ^1^	Patients ^2^	Age ^3^	Gender ^4^
Chronic leukemia (CLL, SLL, T-LGL)	14 (16.5%)	73.5 (46–87)	10 (71.4); 4 (28.6)
Plasma cell diseases (MM)	13 (15.3%)	70 (54–81)	7 (53.8); 6 (46.2)
Acute leukemia (AML, ALL)	7 (8.2%)	72 (40–76)	4 (57.1); 3 (42.9)
Aggressive lymphomas (DLBCL, PTCL, AITL)	17 (20%)	69 (27–87)	10 (58.8); 7 (41.2)
Indolent lymphomas (FL, LPL, NHL, SMZL, CFCL, WM, MCL)	31 (36.5%)	69 (34–92)	20 (64.5); 11 (35.5)
Chronic myeloid neoplasms (PV, MDS)	3 (3.5%)	72 (69–73)	0 (0); 3 (100)
Overall	85 (100%)	70 (27–92)	51 (60); 34 (40)

^1^ Diagnoses included in each group are indicated in parenthesis. ^2^ Number (% of total). ^3^ Median (range). ^4^ Number men (%); number women (%). CLL = chronic lymphocytic leukemia, SLL = small lymphocytic lymphoma, T-LGL = T-Cell large granular lymphocyte leukemia, MM = multiple myeloma, AML = acute myeloid leukemia, ALL = acute lymphoblastic leukemia, DLBCL = diffuse large B-cell lymphoma, PTCL = peripheral T-cell lymphoma, AITL = angioimmunoblastic T-Cell lymphoma, FL = follicular lymphoma, LPL = lymphoplasmacytic lymphoma, NHL = indolent non-Hodgkin B-cell lymphoma not otherwise specified, SMZL = splenic marginal zone lymphoma, CFCL = cutaneous follicle center lymphoma, WM = Waldenström’s macroglobulinemia, MCL = mantle cell lymphoma, PV = Polycythemia Vera, MDS = myelodysplastic syndrome.

**Figure A1:**
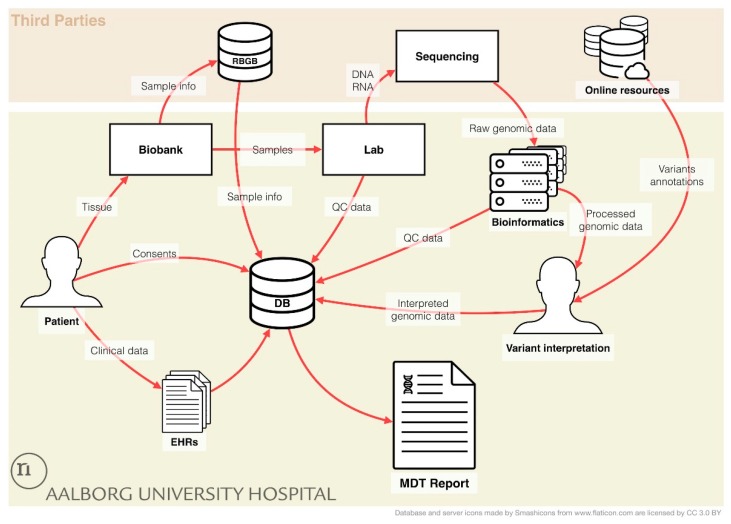
Laboratory Information Management. An overview of the data flow in the ProGen and ProSeq studies. Patient consent is stored in a REDCap database (DB), and clinical data of the patient was from the electronic health records (EHRs) are transferred into the REDCap database. Donated tissue samples are handled and stored in the biobank, and sample information is subsequently placed in the national RBGB database, and automatically imported into the RedCap database, minimizing the time consumption for patient registration. In the laboratory (lab), DNA and RNA is purified from tissue samples or from sorted malignant cells and subsequently sequenced. Raw genomic data is processed using the bioinformatics workflow, and the resulting genomic profile is annotated using online cancer database resources. Following, quality control (QC) data from the Lab and the Bioinformatics analysis is recorded in the REDcap database. Variant interpretation is performed by trained molecular biologists and the resulting interpreted genomic data is transferred to REDCap. Finally, a report for multidisciplinary team conferences (MDT Report) can be generated for each patient.

## Supplementary Materials

The following are available online at https://www.mdpi.com/2072-6694/12/2/312/s1, Figure S1: DNA and yields from MFC sorted samples, Table S1: Variant interpretation results.

## Appendix A. Detailed Materials and Methods

### Appendix A.1. Study Protocols, Screening, and Inclusion

Patients from Department of Haematology, Aalborg University Hospital, Denmark with suspected progression or relapse of hematological malignancy were offered participation in the ProGen protocol. ProGen grants permission to perform single gene analysis on the collected biological material. Eligible for ProGen were patients: Age ≥ 18 years, having suspected relapse or progression of hematologic malignancy, and not receiving relapse treatment at the time of inclusion. Project nurses screened patients, who were hospitalized or had an acute or planned referral through outpatient clinics, for inclusion. Based on clinical information, the patient’s associated clinical hematologist decided, if the identified patient was an eligible candidate for the study. Patients were enrolled in ProGen after signing of informed consent. Biological material for the study and routine diagnostic testing was collected simultaneously.

Patients enrolled in ProGen, who after routine diagnostics were diagnosed with a relapse or progression of a hematologic malignancy, were subsequently offered participation in the ProSeq protocol, which allows broad genomic sequence analysis of tumor DNA of the biological material collected in ProGen. Upon ProSeq enrolment, a patient sample of saliva or a mouth swab was acquired for the sole use as germline DNA. Hence, no germline screenings for cancer predispositions or other genetic diseases were performed. The patient had at least two days from when information about ProSeq was given, to provide informed consent to participate in the study. An assessor could participate in the information process and specific emphasis was put on the risk of incidental findings in the normal DNA, which could reveal an unknown disease predisposition in the host genome [63]. In case of incidental findings, patient and family genetic counselling was warranted by the Department of Clinical Genetics, Aalborg University Hospital. However, the resource was not activated for any of the 85 sequenced patients. A patient could be included in the study at every suspected relapse experienced in the study period.

The ProGen (N-20150042) and ProSeq (N-20160089) studies were approved by the North Denmark Region Committee on Health Research Ethics. ProGen started enrolment of patients 1 February 2016 and ProSeq began enrolment of patients 16 February 2017. Participants gave written informed consent in accordance with the Declaration of Helsinki. Patients enrolled until 31 January 2019 were included in this publication. Both studies were observational and non-interventional studies.

### Appendix A.2. Biobank

Biological material donated included peripheral blood (PB), bone marrow (BM), lymph node biopsies, and saliva or mouth swabs. Methods to purify fractions from PB and BM followed the national guidelines from the Hematological Section of the Danish Cancer Biobank, Bio- and Genome Bank Denmark (RBGB) [64]. In short, PB and BM samples were processed at an in-house biobank within 24 h. BM (2 × 4–5 mL) was aspirated from the iliac crest into EDTA stabilized tubes, and fractions of 2 × 1 mL plasma and 1–8 × 5–10 millions of mononuclear cells (MNC) were isolated. PB was drawn in EDTA stabilized tubes (2 × 9 mL), in serum tubes (1 × 9 mL, no coating or stabilizer), and in cell-free DNA PB collection tubes (1 × 9 mL) (Streck, La Vista, NE, USA). The following fractions were isolated: 2 × 1.5 mL plasma, 1 × 1.5 mL buffy coat, 2 × 1.5 mL serum, and 1–8 × 5–10 millions of MNC. Fractions of plasma, buffy coat, and MNC were isolated using density gradient centrifugation with Ficoll-paque plus solution (GE Healthcare Bio-Science AB, Uppsala, Sweden) and LeucoSep tubes with a membrane (In Vitro, Fredensborg, Denmark). The serum fraction was isolated after centrifugation of the non-stabilized PB sample. MNC fractions were frozen as single cell suspension vital stocks in culture medium containing 10% DMSO. From the cell-free DNA, blood collection tubes 2 × 1.8 mL plasma and 2 × 1.8 mL of the cellular fraction were purified following manufacturer’s descriptions.

Lymph node biopsies were either excised in totos or as an 18 G/1 mm needle ultrasound guided biopsy. One needle biopsy was sent for histopathological evaluation, and one needle biopsy for the study was placed directly in the RNAlater solution, kept for 24–72 h at 4 °C, and subsequently stored at −80 °C. Whole biopsies underwent routine clinical diagnostics tests by an experienced hematopathologist, and excess lymph node material was stored in an RNAlater solution at −80 °C.

Saliva samples were collected in a tube from Oragene DNA (DNA Genotek, Ottawa, Canada), heat inactivated 2 h at 56 °C, and frozen at −20 °C until purification. Mouth swabs were collected using 4 sterile polyester tipped applicators (Puritan Medical Products Company, Guilford, ME, USA) per patient and then tip frozen at −80 °C until purification.

Information on collected biological fractions were stored in the database of RBGB and/or in an in-house REDCap database, Figure A1.

### Appendix A.3. Cell Sorting and DNA/RNA Purification

Multi parameter flow cytometry (MFC) and sorting was used to concentrate the bulk of malignant cell populations when its frequency was below 80%. For phenotype characterization and sorting panel selection, available information from routine clinical diagnostics was used. This included disease status, frequency of malignancy in unpurified fresh tissue, and diagnostic flowcytometric immunophenotyping/immunohistochemical analysis from the fresh analyzed relapsed material (before cryopreservation) or from previous disease stages. If not available, this information was generated by performing an immunophenotyping analysis using selected panels from the “Euroflow antibody panel list” [52] matching the specific disease. A minimal antibody panel was selected for cell enrichment purposes, Table A1. MNC were thawed, DNAse-treated, stained with relevant surface antibodies, washed, and sorted using a FACSAria Cell Sorter (BD Biosciences, San Jose, CA, USA). Sort decision gates were placed to ensure sufficient purity of the malignant cell populations (>80%) without sacrificing malignant subpopulations based on disease heterogeneity (bulk malignancy). Subsequently, the cells were collected, centrifuged, and suspended in lysis buffer for downstream RNA and DNA purification.

**Table A1:** Sorting and staining panels. Minimum sorting panels listing basic targeted antigens as well as optional/custom additions of antigens.

Disease Group	Basic Staining Panel	Optional/Custom Additions *
ALL	CD45/CD34/CD19	CD10, CD20, CD38, IgM, sKappa, sLambda
AML/MDS/PV	CD45/CD34/CD117	HLDAR, CD10, CD13, CD14, CD33 and others
LPL/WM	CD45/CD19/CD20	sKappa, sLambda, IgM, CD5, CD79a
MM	CD45/CD19/CD38	CD138, CD56
SMZL	CD45/CD19/CD20	sKappa, sLambda, IgM, CD22
T-LGLL	CD45/CD5/CD3	CD8, CD4

ALL = acute lymphoblastic leukemia, AML = acute myeloid leukemia, MDS = myelodysplastic syndrome, PV = polycythemia vera, LPL = lymphoplasmacytic lymphoma, WM = Waldenström’s macroglobulinemia, MM = multiple myeloma, SMZL = splenic marginal zone lymphoma, T-LGLL = T-Cell large granular lymphocyte leukemia. * Additional antibodies were selected from the corresponding Euroflow panel.

Lymph node biopsies of maximum 30 mg were homogenized using a Tissuelyser (Qiagen, Germantown, MD, USA). DNA and RNA were purified from MNC, flow sorted malignant cells, or homogenized biopsies using Qiagen’s All Prep DNA/RNA/miRNA kit, following manufacturer’s guidelines. DNA from saliva and mouth swabs were purified using the kits: PrepIT•L2P (DNA Genotek, Ottawa, Canada), and DNeasy Blood & Tissue Kit (Qiagen), respectively, following manufactures guidelines. The quality and quantity of the DNA and RNA were tested using Qubit (Invitrogen, Carlsbad, CA, USA), Bioanalyzer (Agilent, Santa Clara, CA, USA), and Nanodrop (Thermo Scientific, Waltham, MA, USA).

### Appendix A.4. Sequencing

Whole exome sequencing (WES) library preparation was performed using either NEBNext^®^ Ultra™ II DNA Library Prep Kit for Illumina (New England Bio Labs, Ipswich, MA, USA), the Sureselect XT or XT HS Library Prep Kits (Agilent, Santa Clara, CA, USA), the Accel-NGS 2S Hyb DNA Library Kit (SWIFT Biosciences, Ann Arbor, MI, USA), or the Twist Library Preparation EF Kit (TWIST Biosciences, San Francisco, CA, USA). Additional contaminant removal was performed for the majority of samples > 240 ng using the PCR Inhibitor Removal Kit (Zymo Research, Irvine, CA, USA). Exome capture was performed using either the SureSelect XT Clinical Research Exome, the SureSelect XT HS Clinical Research Exome V2 (Agilent), or the Twist Human Core Exome Kit (TWIST Biosciences, San Francisco, CA, USA). For RNAseq library production, TruSeq Stranded mRNA Library Prep Kit (Illumina, San Diego, CA, USA) or NEBNext Ultra II Directional RNA Library Prep Kit for Illumina (New England Biolabs, Ipswich, MA, USA) were used. Sequencing was performed as either 2 × 100 bp or 2 × 150 bp paired-end on either a NextSeq 500 or a NovaSeq 6000 (Illumina, San Diego, CA, USA) producing minimum 26 Gb, 18 Gb, or 33 million reads of raw sequence data for tumor DNA, normal DNA, or tumor RNA samples, respectively.

### Appendix A.5. Bioinformatics Workflow

An in-house workflow for data processing and analysis was developed by integration of several existing programs. Raw FASTQ files were quality trimmed and checked using trimgalore v0.4.3 [65]. Reads were aligned against the GDC GRCh38.d1.vd1 human reference genome sequence using BWA mem v0.7.12 [66] for WES normal and tumor samples and STAR v2.5.3a in 2-pass mode for RNAseq samples. Following marking of duplicates, indel re-alignment, base quality re-calibration, and calculations of general and sequence artefact metrics were performed using GATK v3.8 [53] following the GATK best practices [54,55]. In line with similar studies [36,67], we chose a desired minimum average coverage of 75× and 150× for normal and tumor WES samples, respectively, and 30M sequenced reads for RNAseq libraries. The latter was based on the 2016 ENCODE Experiment Guidelines [68].

For WES data, detection of somatic variants was performed using a combination of Mutect2 v3.8 [69] and Varscan v2.4.1 [70]. Variants marked “PASS” from Mutect2 and “high confidence” variants from Varscan were combined and filtered using a minimum QSS score of 25, a minimum allele frequency (AF) of 0.02, and a minimum allele ratio (AF tumor/AF normal) of 4. Variants were annotated with class, population allele frequencies, sift and polyphen predictions, genomic region and protein domains using the Ensembl variant effect predictor [71]. Following, annotations from the cancer databases OncoKB [22], COSMIC Cancer Gene Census [72], ONgene [73], and CIViC [48] was automatically assigned each variant when applicable. For variant evaluation using Qiagen Clinical Insight Interpret (QCI) (www.qiagenbioinformatics.com), the following criteria had to be met: The variant was exonic ± 10 bp in an exome capture target region. The minimum allele frequency of the alternative allele was ≥10% and supported by ≥ 5 reads. The variant was non-synonymous, and the allele frequency of the variant <5% in any population from 1000 genomes, ESP or gnomAD. For calculation of tumor mutational burden (TMB) a similar filtration was used, only keeping non-synonomus, mis-splicing and indel variants. The total region of interest (ROI) was defined as the sum of exonic ± 2 bp regions within target capture regions. TMB was calculated as number variants/ROI [Mb]. Micro satellite instability (MSI) status was detected using MSISensor v0.2 [74]. Microsatellites in the exome capture target regions with a repeat length ≥ 2 and read coverage in both tumor and normal samples ≥ 20 are examined for instability (FDR threshold = 0.05). The MSI status was given as a percentage of instable sites to the total number of measured sites.

Copy number alterations were inferred using CNVKit 0.9.6 [75]. Off-target bin sizes were estimated from the tumor alignment file and the coverage in these regions was estimated in the tumor and normal samples. Corrections for biases in regional coverage and GC content were performed, and discrete copy number segments from the resulting coverage table were calculated using the CBS segmentation method [76]. Each segment’s absolute integer copy number was derived and filtered, only keeping high-level amplifications (≥ 5 copies) and homozygous deletions (0 copies). Only genes with annotations in either the OncoKB, COSMIC Cancer Gene Census, ONgene, or CIViC database within these segments were reported as either amplified or lost.

Mutational signature analysis was performed using the R package MutationalPatterns V 1.4.3 [77]. The contribution of the COSMIC Mutational Signatures (version 2) [14] to the initial set of somatic variants of each tumor sample was quantified. Mutational signature contributions were grouped as either Deamination of 5−methylcytosine, defective DNA mismatch repair, activation-induced cytidine deaminase activity, polymerase epsilon activity, UV light exposure, tobacco carcinogen exposure, tobacco chewing, alkylating agent association, aristolochic acid exposure, or aflatoxin exposure according to their proposed aetiology.

For RNAseq data, splitting and trimming of reads were performed prior to indel re-alignment. Detection of variants was performed using HaplotypeCaller v3.8 following the GATK best practices. Detection of fusion transcripts (FTs) was performed in the Oshell/OmicSoft Project Environment v10.0.1.29 [78] using both MapFusionReads (MFP) considering the data as single reads and ReportPairedEndFusionGenes (RPEFG) utilizing the paired-end information. Minimal read coverage was set to 5 and the minimal distance between potential fusion genes was set to 5000 bp. Due to a high false positive rate especially using RPEFG, and a high amount of non-informative FTs, further filtering was made. Both gene partners must have an approved HGNC symbol, and FTs were filtered if they did not have an entry in either COSMIC fusion genes, TICdb [79], or Atlasgeneticsoncology.org [80] or if at least one of the fusion partners had and entry in either the OncoKB, COSMIC Cancer Gene Census, ONgene, or CIViC database.

### Appendix A.6. Variant Classification and Evaluation

The filtered variant call file (VCF), along with patient information (age, gender, and diagnosis) and information regarding detected fusion transcripts, gene losses, and gains were uploaded to the decision support tool QCI for variant evaluation. Filtered variants were on the basis of their therapeutic, prognostic or diagnostic potential automatically classified into the following groups using the 2017 AMP/ASCO/CAP tier system [16]: Variants with strong clinical significance (tier 1A and 1B), variants with potential clinical significance (tier 2C and 2D), variants of unknown clinical significance (tier 3), or benign or likely benign variants (tier 4). Based on clinical guidelines and information from clinicaltrials.gov, guideline recommended therapies and clinical trials were associated to variants by QCI, when available. Two trained molecular biologists manually investigated or verified the clinical significance of all potentially informative variants. These included all fusion transcripts, gain or loss CNA, and all SNV automatic classified as pathogenic, actionable, or prognostic by QCI or variants with an entry in either the OncoKB, COSMIC Cancer Gene Census, ONGene, or CIViC databases. All variants found to be reportable were manually verified by visual inspection of supporting reads in the WES data, and if possible in the RNAseq data in IGV, Table S1 [81]. A report of reportable variants could subsequently be generated for presentation at multidisciplinary team conferences.

### Appendix A.7. Laboratory Information Management Systems

In order to allow a proper workflow monitoring, a Laboratory Information Management System (LIMS) was developed. The system utilizes REDCap [61,62] to store the workflow metadata from inclusions of patients to the final results of the genomic analysis, which can be exported to a multidisciplinary teams report, Figure A1. The REDCap part encompasses three projects: Patient inclusion focusing on patient’s consents and tissue sampling, laboratory management recording the various handling steps in the laboratory, and clinical data recording the patient’s clinical history related to their hematological malignancy containing information about treatment, laboratory test results, comorbidities, medication, etc. A web platform was developed to aggregate the information from the three REDCap databases, hereby circumventing limitations for the implementation of a LIMS solution. This platform enabled monitorization and validation of the quality of the data, Figure A1. The platform utilizes the REDCap Application Programming Interface (API) for a seamless integration and a tool was integrated to upload information on biological fractions stored in the RBGB database.
