# Supplementary material for: Development of a Precision Medicine Workflow in Hematological Cancers, Aalborg University Hospital, Denmark
Source: Cancers (Basel). 2020 Jan 29;12(2):312. doi: 10.3390/cancers12020312 (PMC7073219; doi:10.3390/cancers12020312)
Supplement: Supplementary file 1 [file cancers-12-00312-s001.pdf]

Supplementary Material

Boxplots of DNA and RNA Purification yields from MFC sorted samples grouped by patient group or sample type are shown in Figure S1.

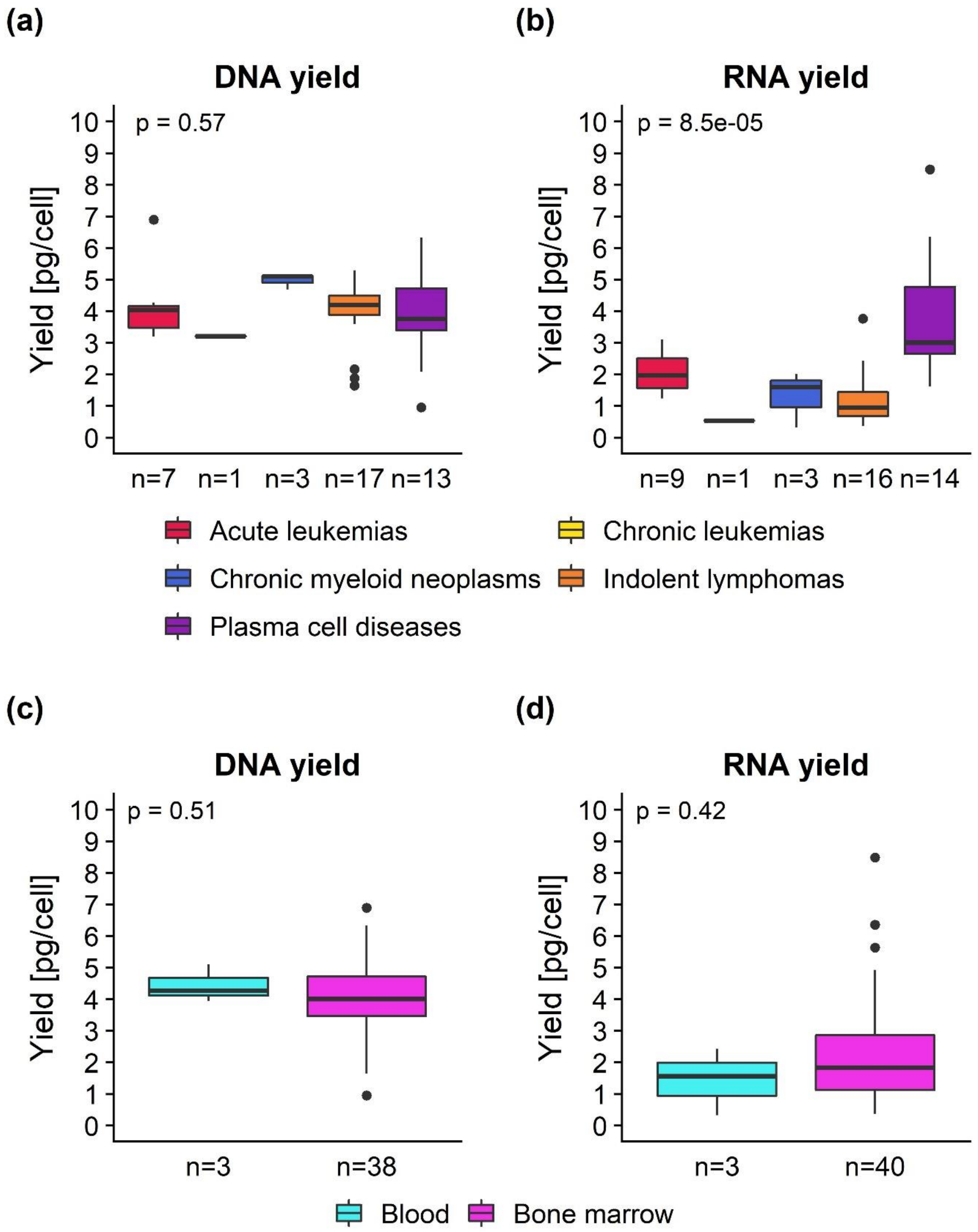

Figure S1. DNA (panels (**a**) and (**c**)) and RNA (panels (**b**) and (**d**)) yields from MFC sorted samples divided into different patient groups (panels (**a**) and (**b**)) or sample types (panels (**c**) and (**d**)). The p-values indicate, whether the mean values are equal among all groups (by one-way ANOVA). If multiple fractions were collected after sorting, only the sequenced one is represented in the figure.

Table S1. Variant interpretation results. The highest clinical assessment in each of the three categories; overall, therapeutic, and prognostic and the corresponding gene(s) of each sample subjected to variant interpretation is given. The MSI status and TMB for each sample is also shown. NA = Sample did not have a gene with a clinical relevance ≥ 2. Explanation of each Criteria ID is given in the sheet Value explanation. * TMB is given as no. mutations/Mb. ** MSI status is given as percentage of instable microsatellites.

Patient ID	Diagnosis	Diagnosis Group	Highest assessment	Genes providing highest assessment	Highest therapeutic assessment	Therapeutic significant genes	Highest prognostic assessment	Prognostic significant genes	TMB *	MSI [%] **
005-1	AML	Acute leukemias	1A	RUNX1	2C-R2	RUNX1	1A-P	RUNX1	0.179	0.44
076-1	AML	Acute leukemias	1A	IDH2 NPM1 DNMT3A	1A-S1	IDH2	1A-P	IDH2 NPM1 DNMT3A	0.864	1.01
144-1	AML	Acute leukemias	2D	NUP98-NSD1	NA	NA	NA	NA	0	0.65
156-2	AML	Acute leukemias	3	PLAGL2 EEF1A1-TYK2 BCL2L1	NA	NA	NA	NA	0.215	1.24
239-1	AML	Acute leukemias	1A	TP53	1A-S2	TP53	1B-P	TP53	0.564	0.2
298-1	AML	Acute leukemias	1A	DNMT3A IDH1	1A-S1	IDH1	1A-P	DNMT3A IDH1	0.488	0.06
349-1	ALL	Acute leukemias	3	PRDM2 NT5C2 TSC2	NA	NA	NA	NA	1.751	1.38
001-1	CLL	Chronic leukemias	1A	TP53	1A-S1	TP53	1A-P	TP53	0.564	0
002-1	CLL	Chronic leukemias	3	BCOR B2M-CXCR4 FBXW7	NA	NA	NA	NA	1	0
107-1	CLL	Chronic leukemias	2C	TET2	2C-S2 2C-R2	TET2	NA	NA	0.511	0.01
124-1	CLL	Chronic leukemias	3	PIM3-SCO2 CIITA-DEXI	NA	NA	NA	NA	0.073	0.01
136-1	CLL	Chronic leukemias	3	CIITA-DEXI EGR2 FBXW7	NA	NA	NA	NA	0.601	0
138-1	CLL	Chronic leukemias	1A	NOTCH1 TP53	1A-S1	TP53	1A-P	NOTCH1 TP53	1.131	0

147-1	CLL	Chronic leukemias	3	RPS15	NA	NA	NA	NA	0.62	0
158-1	CLL	Chronic leukemias	1A	TP53 SF3B1	1A-S1	TP53	1A-P	TP53 SF3B1	0.656	0.01
164-1	CLL	Chronic leukemias	3	PTPN13	NA	NA	NA	NA	0.693	0
168-1	SLL	Chronic leukemias	2C	KMT2D NOTCH1	2C-R2	NOTCH1	NA	NA	0.693	0
234-1	CLL	Chronic leukemias	2C	ATM	2C-CT	ATM	NA	NA	0.839	0.09
249-1	CLL	Chronic leukemias	3	ACTB-GNAS CIITA-DEXI	NA	NA	NA	NA	0.036	0
383-1	T-LGLL	Chronic leukemias	3	IGF1R STAT3 KMT2A TCF25-ANKRD11	NA	NA	NA	NA	0.753	0.33
261-1	PV	Chronic myeloid neoplasms	3	INSR	NA	NA	NA	NA	0.072	0.17
287-1	MDS	Chronic myeloid neoplasms	NA	NA	NA	NA	NA	NA	0.072	0.04
397-1	MDS	Chronic myeloid neoplasms	1A	RUNX1	2C-R2	RUNX1	1A-P	RUNX1	0.681	0.2
015-1	DLBCL	Aggressive lymphomas	2C	EZH2	2C-R2	EZH2	NA	NA	2.553	0
019-1	DLBCL	Aggressive lymphomas	2C	ATM EZH2	2C-R2	EZH2	NA	NA	1.24	0.05
087-1	DLBCL	Aggressive lymphomas	3	INSL3-JAK3 RCC1-IRF4 ACTB-GNAS	NA	NA	NA	NA	0.036	0.16
123-1	DLBCL	Aggressive lymphomas	2C	CREBBP EZH2	2C-R2	EZH2	NA	NA	2.881	0.09
123-2	DLBCL	Aggressive lymphomas	2C	CREBBP EZH2	2C-R2	EZH2	NA	NA	3.465	0.16

155-1	DLBCL	Aggressive lymphomas	3	ARID1B AFF3-MAPKAP1 TP53	NA	NA	NA	NA	0.766	0.03
155-2	DLBCL	Aggressive lymphomas	2C	MYC	NA	NA	NA	NA	1.183	0.52
173-1	DLBCL	Aggressive lymphomas	2C	ATM	2C-CT	ATM	NA	NA	1.058	0.15
183-1	PTCL	Aggressive lymphomas	2C	MBNL1-BCL6	NA	NA	NA	NA	0.656	0.01
189-1	DLBCL	Aggressive lymphomas	2C	KMT2D TP53	2C-S1	TP53	NA	NA	2.261	0.5
196-1	DLBCL	Aggressive lymphomas	2C	MYD88	2C-CT	MYD88	NA	NA	3.83	0.33
292-1	DLBCL	Aggressive lymphomas	2C	MYD88	2C-CT	MYD88	NA	NA	1.721	0.4
316-1	PTCL	Aggressive lymphomas	NA	NA	NA	NA	NA	NA	0	0.14
321-1	DLBCL	Aggressive lymphomas	2C	SRSF2 CREBBP KMT2D	2C-R2	SRSF2	NA	NA	0.985	0.05
321-2	DLBCL	Aggressive lymphomas	2C	KMT2D CREBBP	2C-CT	KMT2D CREBBP	NA	NA	1.714	0.39
333-1	DLBCL	Aggressive lymphomas	2C	CDKN2A KMT2D CIITA-BCL6	2C-CT	CDKN2A KMT2D	NA	NA	3.55	0.35
333-2	DLBCL	Aggressive lymphomas	2C	KMT2D CDKN2A CIITA-BCL6	2C-CT	KMT2D CDKN2A	NA	NA	3.515	0.65
404-1	DLBCL	Aggressive lymphomas	2C	MYD88 KMT2D	2C-CT	MYD88 KMT2D	NA	NA	2.654	0.79
407-1	DLBCL	Aggressive lymphomas	2C	KRAS FBXW7	2C-R1	KRAS	2D-P	BIRC3	4.339	0.17
437-1	DLBCL	Aggressive lymphomas	3	MAPK1 LYN GAD2 KDR BTG1 BIRC6 NCOR1 AR	NA	NA	NA	NA	1.542	0
490-1	DLBCL	Aggressive lymphomas	2D	TNFRSF14	NA	NA	2D-P	TNFRSF14	4.088	0.78
503-1	AITL	Aggressive lymphomas	2C	TET2	2C-S2 2C-R2	TET2	NA	NA	0.215	0.03

024-1	FL	Indolent lymphomas	2C	LRP1B EZH2	2C-R1	LRP1B	NA	NA	3.55	1.01
026-1	FL	Indolent lymphomas	2C	KMT2D CREBBP	2C-CT	KMT2D CREBBP	NA	NA	1.004	0.18
028-1	FL	Indolent lymphomas	NA	NA	NA	NA	NA	NA	0.108	0
032-1	LPL	Indolent lymphomas	2C	MYD88	2C-CT	MYD88	NA	NA	0.717	0.58
033-1	NHL	Indolent lymphomas	2C	TP53	2C-S1	TP53	2D-P	TP53	0.753	0.08
099-1	LPL	Indolent lymphomas	2C	MYD88 ARID1A CXCR4	2C-CT	MYD88 ARID1A CXCR4	NA	NA	0.789	0.35
117-1	FL	Indolent lymphomas	2C	EZH2 CREBBP KMT2D	2C-R2	EZH2	NA	NA	0.488	0
133-1	NHL	Indolent lymphomas	3	ARAF CD74-RALYL CLK1-CXCR4	NA	NA	NA	NA	1	0.05
160-1	FL	Indolent lymphomas	2C	TP53 KMT2D CREBBP SRSF2	2C-S1	TP53	NA	NA	1.148	0.4
184-1	SMZL	Indolent lymphomas	2C	ATM CDKN2A	2C-CT	ATM CDKN2A	NA	NA	0.968	0.82
206-1	SMZL	Indolent lymphomas	2C	MYD88	2C-CT	MYD88	NA	NA	0.729	0
217-1	NHL	Indolent lymphomas	2C	TP53	2C-S1	TP53	2D-P	TP53	0.948	0.03
222-1	LPL	Indolent lymphomas	2C	MYD88	2C-CT	MYD88	NA	NA	0.656	0
230-1	NHL	Indolent lymphomas	NA	NA	NA	NA	NA	NA	0	0
240-1	FL	Indolent lymphomas	2C	KMT2D EZH2	2C-R2	EZH2	NA	NA	2.69	0.14
248-1	NHL	Indolent lymphomas	2C	TP53	2C-S1	TP53	2D-P	TP53	0.547	0
258-1	CFCL	Indolent lymphomas	2C	EP300 EZH2	2C-R2	EZH2	NA	NA	1.865	0.29

277-1	FL	Indolent lymphomas	2C	KMT2D MTAP	2C-CT	KMT2D	NA	NA	1.47	0.41
299-1	FL	Indolent lymphomas	2C	EZH2 KMT2D	2C-R2	EZH2	NA	NA	0.861	0
317-1	FL	Indolent lymphomas	2C	KMT2D CREBBP	2C-CT	KMT2D CREBBP	NA	NA	1.578	0.04
321-3	FL	Indolent lymphomas	2C	KMT2D SRSF2 CREBBP	2C-R2	SRSF2	NA	NA	2.626	0.43
350-1	NHL	Indolent lymphomas	3	KLF2 REL PTPRD BCL11A	NA	NA	NA	NA	0.61	0.11
412-1	NHL	Indolent lymphomas	2C	MYD88	2C-CT	MYD88	NA	NA	1.112	0.08
425-1	LPL	Indolent lymphomas	2C	MYD88 CXCR4	2C-CT	MYD88 CXCR4	NA	NA	1.004	0.05
435-1	WM	Indolent lymphomas	2C	MYD88	2C-CT	MYD88	NA	NA	1.04	0.19
438-1	NHL	Indolent lymphomas	2C	TP53	2C-S1	TP53	2D-P	TP53	0.143	0
439-1	MCL	Indolent lymphomas	2C	ATM CDKN2A MYD88 CCND1 NRAS LRP1B	2C-R1	NRAS	NA	NA	1.399	0.86
448-1	NHL	Indolent lymphomas	2C	EP300	2C-CT	EP300	NA	NA	1.757	0.32
454-1	WM	Indolent lymphomas	2C	MYD88	2C-CT	MYD88	NA	NA	0.789	0.94
458-1	LPL	Indolent lymphomas	2C	CXCR4 MYD88	2C-CT	CXCR4 MYD88	NA	NA	0.215	0.02
466-1	MCL	Indolent lymphomas	2C	TP53	2C-S1	TP53	2D-P	TP53	1.459	0.05
003-1	MM	Plasma cell diseases	2C	NRAS	2C-R1	NRAS	NA	NA	1.202	0.01
023-1	MM	Plasma cell diseases	3	BARD1 OLAH NFKB2 FGF8 B2M-ELK2AP BRDT	NA	NA	NA	NA	2.845	0.3

027-1	MM	Plasma cell diseases	3	TBC1D13-BCL7A NFKB2	NA	NA	NA	NA	0.864	0.21
085-1	MM	Plasma cell diseases	2C	BRAF	2C-S1	BRAF	NA	NA	1.841	0.53
115-1	MM	Plasma cell diseases	2C	NRAS NTRK3	2C-R1	NRAS	NA	NA	4.396	0.94
127-1	MM	Plasma cell diseases	2C	KRAS BRAF	2C-R1	KRAS	NA	NA	1.901	0.37
135-1	MM	Plasma cell diseases	2C	KRAS	2C-R1	KRAS	NA	NA	3.622	0.31
156-1	MM	Plasma cell diseases	2C	NRAS	2C-R1	NRAS	NA	NA	2.735	0.17
159-1	MM	Plasma cell diseases	2C	KRAS	2C-R1	KRAS	NA	NA	1.969	0.1
221-1	MM	Plasma cell diseases	3	PREX2 IRS4	NA	NA	NA	NA	0.584	0.26
228-1	MM	Plasma cell diseases	3	CCND1 CLIP1 CDH1 CDKN1A	NA	NA	NA	NA	1.349	0.01
365-1	MM	Plasma cell diseases	2C	TP53	2C-S1	TP53	NA	NA	3.012	1.5
384-1	MM	Plasma cell diseases	3	IKBKB ALPP	NA	NA	NA	NA	0.323	0.05
453-1	MM	Plasma cell diseases	2C	CUX1-BRAF	NA	NA	NA	NA	2.188	0.51
453-2	MM	Plasma cell diseases	2C	CUX1-BRAF	NA	NA	NA	NA	2.48	0.25

1A-S1 = Biomarker predicts a response to FDA, EMA or PMDA approved therapies for this diagnosis, 1A-S2 = Biomarker included in professional guidelines is predictive of response to FDA, EMA or PMDA approved therapies for this diagnosis, 1A-R1 = Biomarker predicts resistance to FDA, EMA or PMDA approved therapies for this diagnosis, 1A-R2 = Biomarker included in professional guidelines is predictive of resistance to FDA, EMA or PMDA approved therapies for this diagnosis, 1B-S = Biomarker predicts a response to therapies for this diagnosis based on well-powered studies, 1B-R = Biomarker predicts resistance to therapies for this diagnosis based on well-powered studies, 1A-P = Biomarker included in professional guidelines is prognostic for this diagnosis, 1B-P = Biomarker is prognostic for this diagnosis based on well-powered studies, 1A-D = Biomarker included in professional guidelines is diagnostic for this diagnosis, 1B-D = Biomarker is diagnostic for this diagnosis based on well-powered studies, 2C-S1 = Biomarker is associated with response to FDA, EMA or PMDA approved therapies for a different diagnosis, 2C-S2 = Biomarker included in professional guidelines is associated with response to FDA, EMA or PMDA approved therapies for a different diagnosis, 2C-R1 = Biomarker is associated with resistance to FDA, EMA or PMDA approved therapies for a different diagnosis, 2C-R2 = Biomarker included in

professional guidelines is associated with resistance to FDA, EMA or PMDA approved therapies for a different diagnosis, 2C-CT = Biomarker serves as an inclusion criterion for one or more clinical trials, 2D-S = Biomarker shows plausible response based on case studies, 2D-R = Biomarker shows plausible response based on case studies, 2C-P = Biomarker is prognostic based on multiple small studies, 2D-P = Biomarker is plausibly prognostic for this diagnosis based on case studies, 2C-D = Biomarker is diagnostic based on multiple small studies, 2D-D = Biomarker is plausibly diagnostic for this diagnosis based on case studies, 3 = Biomarker has uncertain clinical significance and not known to be likely benign or benign, 4 = Biomarker is classified as likely benign based on the ACMG/AMP guidelines.
